# Targeted enrichment of novel chloroplast-based probes reveals a large-scale phylogeny of 412 bamboos

**DOI:** 10.1186/s12870-020-02779-5

**Published:** 2021-02-05

**Authors:** Jiongliang Wang, Weixue Mu, Ting Yang, Yue Song, Yin Guang Hou, Yu Wang, Zhimin Gao, Xin Liu, Huan Liu, Hansheng Zhao

**Affiliations:** 1 Key Laboratory of National Forestry and Grassland Administration/Beijing for Bamboo & Rattan Science and Technology, Beijing, China; 2 Institute of Gene Science and Industrialization for Bamboo and Rattan Resources, International Center for Bamboo and Rattan, Beijing, China; 3 State Key Laboratory of Agricultural Genomics, BGI-Shenzhen, Shenzhen, China

**Keywords:** Bambusoideae, Chloroplast, Probe, Targeted enrichment, Bamboo phylogeny

## Abstract

**Background:**

The subfamily Bambusoideae belongs to the grass family Poaceae and has significant roles in culture, economy, and ecology. However, the phylogenetic relationships based on large-scale chloroplast genomes (CpGenomes) were elusive. Moreover, most of the chloroplast DNA sequencing methods cannot meet the requirements of large-scale CpGenome sequencing, which greatly limits and impedes the in-depth research of plant genetics and evolution.

**Results:**

To develop a set of bamboo probes, we used 99 high-quality CpGenomes with 6 bamboo CpGenomes as representative species for the probe design, and assembled 15 M unique sequences as the final pan-chloroplast genome. A total of 180,519 probes for chloroplast DNA fragments were designed and synthesized by a novel hybridization-based targeted enrichment approach. Another 468 CpGenomes were selected as test data to verify the quality of the newly synthesized probes and the efficiency of the probes for chloroplast capture. We then successfully applied the probes to synthesize, enrich, and assemble 358 non-redundant CpGenomes of woody bamboo in China. Evaluation analysis showed the probes may be applicable to chloroplasts in Magnoliales, Pinales, Poales et al. Moreover, we reconstructed a phylogenetic tree of 412 bamboos (358 in-house and 54 published), supporting a non-monophyletic lineage of the genus *Phyllostachys*. Additionally, we shared our data by uploading a dataset of bamboo CpGenome into CNGB (https://db.cngb.org/search/project/CNP0000502/) to enrich resources and promote the development of bamboo phylogenetics.

**Conclusions:**

The development of the CpGenome enrichment pipeline and its performance on bamboos recommended an inexpensive, high-throughput, time-saving and efficient CpGenome sequencing strategy, which can be applied to facilitate the phylogenetics analysis of most green plants.

**Supplementary Information:**

The online version contains supplementary material available at 10.1186/s12870-020-02779-5.

## Background

The subfamily Bambusoideae belongs to the grass family Poaceae and exhibits substantial phenotypic diversity, with 1642 species in 125 genera, three tribes, and 15 subtribes, which have been classified into ~ 75 clades [1]. The Bambuseae consists of tropical woody bamboos (Bambuseae), temperate woody bamboos (Arundinarieae) and herbaceous bamboo tribe (Olyreae). Bambusoideae predominantly distributed in the Old World, such as China, Japan, Thailand, Indonesia, and the countries of Southeast Asian. As one of the most ecologically and industrially valuable tribes of Bambusoideae, woody bamboos were used for furniture, paper, fiber textiles, and fuel [2]. In total, about 500 bamboos are distributed in Asia, spanning a wide geographic and temperature range. However, infrequent, incongruent, and unpredictable flowering events as well as unstable vegetative characteristics, severely restricted the identification and classification of woody bamboos. The phylogenetic relationships based on more massive amounts of woody bamboos remain elusive due to the lack of extensive and high-quality genomic resources.

The chloroplast genome (CpGenome) is an essential resource for the study of plant evolution [3]. This organelle is one of the most technically accessible regions of the genome. The chloroplast genomic DNA of green plants commonly exhibits a conserved genome structure that contains two copies of inverted repeat (IR) separating the small single-copy region (SSC) and the large single-copy region (LSC) [2, 4, 5]. The CpGenome has been a popular source of reconstructing the phylogeny of green plants, and many chloroplast DNA loci are contributing to the development of plant taxonomy. To obtain chloroplast DNA suitable for whole chloroplast genome sequencing, it can be traditionally enriched by using the sucrose gradient centrifugation method [6], the high salt method [7], long PCR technology by using primers [8]. The characters of the strategies above are the use of physical methods to extract chloroplast DNA or the need for high quality, sufficiently extracted cellar DNA and the appropriate primers. With the development of sequencing technology, next-generation sequencing (NGS) has the advantageous characteristics of high-throughput and efficient, resulting in a rapid increase in the amount of sequencing data. Chloroplast DNA generally accounts for only about 0.5–13% of the whole genome [9]. But, the chloroplast DNA sequencing data from the whole genome sequencing (WGS) data produced a lot of “useless” data except for “useful” ones, consuming much of the sequencing capacity and reducing the efficiency of parallelly chloroplast sequencing. The above methods for obtaining chloroplast DNA sequencing data cannot meet the needs of large-scale CpGenome sequencing, which significantly restricts and hinders the in-depth research of plant genetics and evolution.

In this study, the main goals were: (1) To develop and evaluate a pipeline to target-enrich and assembly the chloroplast data of bamboos. (2) To obtain high-quality and high coverage of bamboo CpGenomes by the pipeline, to reconstruct a phylogenetic tree, and to promote phylogenetic knowledge of bamboo. (3) To share the new sequenced bamboo CpGenomes, allowing researchers to quickly compare suspect chloroplast data and explore the bamboo CpGenomes.

## Methods

### Species selection for probe design and evaluation

To improve the variability and versatility of the probes, we selected 567 representative species from the 3654 published CpGenomes species (collected from NCBI, Released Dec 2018) to design and evaluate probes for a targeted enrichment strategy of CpGenomes (Supplementary Table S1 and S2). Among the 567 species, 22 are bamboo species. For data preprocessing, we elucidated our approach in a flow chart (Supplementary Figure S1). A phylogenetic tree (Supplementary Figure S2) was constructed based on the 567 complete CpGenomes, which spanned the phylogenetic diversity of 7 major clades, including 40 orders and 57 families. The model species in each clade were selected as core candidates. Thus, a total of 99 CpGenomes, including 6 bamboo CpGenomes, were chosen as the representative species for the probe design (Table 1), and the remaining (468 CpGenomes) were chosen as test data further to assess the efficiency of the probes for chloroplast capture. The species for probe design and the species for probe evaluation were different genera but belong to the same family (e.g., *Danthonia* and *Chionochloa*, both are Poaceae).

**Table 1:** The taxonomic composition of the chloroplast genome sequences which used for design probes

NCBI species ID	Classification	Order	Family	Genus	Species
NC_010093.1	Monocots	*Acorales*	*Acoraceae*	*Acorus*	*Acorus americanus*
NC_022133.1	Monocots	*Poales*	*Poaceae*	*Aegilops*	*Aegilops tauschii*
NC_023934.1	Monocots	*Poales*	*Poaceae*	*Arundinaria*	*Arundinaria appalachiana*
NC_012927.1	Monocots	*Poales*	*Poaceae*	*Bambusa*	*Bambusa oldhamii*
NC_011032.1	Monocots	*Poales*	*Poaceae*	*Brachypodium*	*Brachypodium distachyon*
NC_025663.1	Monocots	*Asparagales*	*Orchidaceae*	*Corallorhiza*	*Corallorhiza wisteriana*
NC_021432.1	Monocots	*Asparagales*	*Orchidaceae*	*Cymbidium*	*Cymbidium tracyanum*
NC_025232.1	Monocots	*Poales*	*Poaceae*	*Danthonia*	*Danthonia californica*
NC_009601.1	Monocots	*Dioscoreales*	*Dioscoreaceae*	*Dioscorea*	*Dioscorea elephantipes*
NC_024715.1	Monocots	*Poales*	*Poaceae*	*Fargesia*	*Fargesia nitida*
NC_019648.1	Monocots	*Poales*	*Poaceae*	*Festuca*	*Festuca altissima*
NC_024728.1	Monocots	*Liliales*	*Liliaceae*	*Fritillaria*	*Fritillaria cirrhosa*
NC_024720.1	Monocots	*Poales*	*Poaceae*	*Indocalamus*	*Indocalamus wilsonii*
NC_022926.1	Monocots	*Zingiberales*	*Musaceae*	*Musa*	*Musa textilis*
NC_001320.1	Monocots	*Poales*	*Poaceae*	*Oryza*	*Oryza sativa Japonica*
NC_017609.1	Monocots	*Asparagales*	*Orchidaceae*	*Phalaenopsis*	*Phalaenopsis equestris*
NC_023245.1	Monocots	*Poales*	*Poaceae*	*Pharus*	*Pharus lappulaceus*
NC_013991.2	Monocots	*Arecales*	*Arecaceae*	*Phoenix*	*Phoenix dactylifera*
NC_015817.1	Monocots	*Poales*	*Poaceae*	*Phyllostachys*	*Phyllostachys edulis*
NC_022850.1	Monocots	*Poales*	*Poaceae*	*Setaria*	*Setaria italica*
NC_008602.1	Monocots	*Poales*	*Poaceae*	*Sorghum*	*Sorghum bicolor*
NC_002762.1	Monocots	*Poales*	*Poaceae*	*Triticum*	*Triticum aestivum*
NC_015894.1	Monocots	*Alismatales*	*Araceae*	*Wolffiella*	*Wolffiella lingulata*
NC_024725.1	Monocots	*Poales*	*Poaceae*	*Yushania*	*Yushania levigata*
NC_001666.2	Monocots	*Poales*	*Poaceae*	*Zea*	*Zea mays*
NC_005086.1	Basal angiosperms	*Amborellales*	*Amborellaceae*	*Amborella*	*Amborella trichopoda*
NC_006050.1	Basal angiosperms	*Nymphaeales*	*Nymphaeaceae*	*Nymphaea*	*Nymphaea alba*
NC_023242.1	Magnoliidae	*Magnoliales*	*Magnoliaceae*	*Magnolia*	*Magnolia sprengeri*
NC_008457.1	Magnoliidae	*Piperales*	*Piperaceae*	*Piper*	*Piper cenocladum*
NC_026690.1	Eudicots	*Ericales*	*Actinidiaceae*	*Actinidia*	*Actinidia chinensis*
NC_009265.1	Eudicots	*Brassicales*	*Brassicaceae*	*Aethionema*	*Aethionema cordifolium*
NC_015621.1	Eudicots	*Asterales*	*Asteraceae*	*Ageratina*	*Ageratina adenophora*
NC_022412.1	Eudicots	*Myrtales*	*Myrtaceae*	*Angophora*	*Angophora costata*
NC_000932.1	Eudicots	*Brassicales*	*Brassicaceae*	*Arabidopsis*	*Arabidopsis thaliana*
NC_009268.1	Eudicots	*Brassicales*	*Brassicaceae*	*Arabis*	*Arabis hirsuta*
NC_022810.1	Eudicots	*Apiales*	*Araliaceae*	*Aralia*	*Aralia undulata*
NC_021121.1	Eudicots	*Ericales*	*Primulaceae*	*Ardisia*	*Ardisia polysticta*
NC_025910.1	Eudicots	*Asterales*	*Asteraceae*	*Artemisia*	*Artemisia montana*
NC_022432.1	Eudicots	*Gentianales*	*Asclepiadaceae*	*Asclepias*	*Asclepias syriaca*
NC_016734.1	Eudicots	*Brassicales*	*Brassicaceae*	*Brassica*	*Brassica napus*
NC_024541.1	Eudicots	*Ericales*	*Theaceae*	*Camellia*	*Camellia crapnelliana*
NC_010323.1	Eudicots	*Brassicales*	*Caricaceae*	*Carica*	*Carica papaya*
NC_014674.1	Eudicots	*Fagales*	*Fagaceae*	*Castanea*	*Castanea mollissima*
NC_011163.1	Eudicots	*Fagales*	*Fagaceae*	*Cicer*	*Cicer arietinum*
NC_025642.1	Eudicots	*Lamiales*	*Orobanchaceae*	*Cistanche*	*Cistanche phelypaea*
NC_008334.1	Eudicots	*Sapindales*	*Rutaceae*	*Citrus*	*Citrus sinensis*
NC_008535.1	Eudicots	*Gentianales*	*Rubiaceae*	*Coffea*	*Coffea arabica*
NC_022409.1	Eudicots	*Myrtales*	*Myrtaceae*	*Corymbia*	*Corymbia eximia*
NC_007144.1	Eudicots	*Cucurbitales*	*Cucurbitaceae*	*Cucumis*	*Cucumis sativus*
NC_009963.1	Eudicots	*Solanales*	*Convolvulaceae*	*Cuscuta*	*Cuscuta exaltata*
NC_014569.1	Eudicots	*Geraniales*	*Geraniaceae*	*Erodium*	*Erodium texanum*
NC_022396.1	Eudicots	*Myrtales*	*Myrtaceae*	*Eucalyptus*	*Eucalyptus aromaphloia*
NC_015206.1	Eudicots	*Rosales*	*Rosaceae*	*Fragaria*	*Fragaria vesca*
NC_007942.1	Eudicots	*Fabales*	*Fabaceae*	*Glycine*	*Glycine max*
NC_016668.1	Eudicots	*Malvales*	*Malvaceae*	*Gossypium*	*Gossypium raimondii*
NC_024732.1	Eudicots	*Asterales*	*Campanulaceae*	*Hanabusaya*	*Hanabusaya asiatica*
NC_023110.1	Eudicots	*Asterales*	*Asteraceae*	*Helianthus*	*Helianthus decapetalus*
NC_026726.1	Eudicots	*Solanales*	*Solanaceae*	*Iochroma*	*Iochroma loxense*
NC_009808.1	Eudicots	*Solanales*	*Convolvulaceae*	*Ipomoea*	*Ipomoea purpurea*
NC_026677.1	Eudicots	*Fabales*	*Fabaceae*	*Libidibia*	*Libidibia coriaria*
NC_024064.1	Eudicots	*Malpighiales*	*Chrysobalanaceae*	*Licania*	*Licania alba*
NC_002694.1	Eudicots	*Fabales*	*Fabaceae*	*Lotus*	*Lotus japonicus*
NC_023090.1	Eudicots	*Fabales*	*Fabaceae*	*Lupinus*	*Lupinus luteus*
NC_010433.1	Eudicots	*Malpighiales*	*Euphorbiaceae*	*Manihot*	*Manihot esculenta*
NC_003119.6	Eudicots	*Fabales*	*Fabaceae*	*Medicago*	*Medicago truncatula*
NC_012615.1	Eudicots	*Ranunculales*	*Ranunculaceae*	*Megaleranthis*	*Megaleranthis saniculifolia*
NC_008359.1	Eudicots	*Rosales*	*Moraceae*	*Morus*	*Morus indica*
NC_025339.1	Eudicots	*Proteales*	*Nelumbonaceae*	*Nelumbo*	*Nelumbo nucifera*
NC_010358.1	Eudicots	*Myrtales*	*Onagraceae*	*Oenothera*	*Oenothera argillicola*
NC_013707.2	Eudicots	*Lamiales*	*Oleaceae*	*Olea*	*Olea europaea*
NC_006290.1	Eudicots	*Apiales*	*Araliaceae*	*Panax*	*Panax ginseng*
NC_009259.1	Eudicots	*Fabales*	*Fabaceae*	*Phaseolus*	*Phaseolus vulgaris*
NC_009143.1	Eudicots	*Malpighiales*	*Salicaceae*	*Populus*	*Populus trichocarpa*
NC_014697.1	Eudicots	*Rosales*	*Rosaceae*	*Prunus*	*Prunus persica*
NC_015996.1	Eudicots	*Rosales*	*Rosaceae*	*Pyrus*	*Pyrus pyrifolia*
NC_016736.1	Eudicots	*Malpighiales*	*Euphorbiaceae*	*Ricinus*	*Ricinus communis*
NC_026722.1	Eudicots	*Malpighiales*	*Salicaceae*	*Salix*	*Salix purpurea*
NC_026202.1	Eudicots	*Lamiales*	*Scrophulariaceae*	*Scrophularia*	*Scrophularia takesimensis*
NC_023085.1	Eudicots	*Saxifragales*	*Crassulaceae*	*Sedum*	*Sedum sarmentosum*
NC_016730.1	Eudicots	*Caryophyllales*	*Caryophyllaceae*	*Silene*	*Silene latifolia*
NC_008096.2	Eudicots	*Solanales*	*Solanaceae*	*Solanum*	*Solanum tuberosum*
NC_014676.2	Eudicots	*Malvales*	*Malvaceae*	*Theobroma*	*Theobroma cacao*
NC_024034.1	Eudicots	*Fabales*	*Fabaceae*	*Trifolium*	*Trifolium grandiflorum*
NC_021449.1	Eudicots	*Lamiales*	*Lentibulariaceae*	*Utricularia*	*Utricularia gibba*
NC_021091.1	Eudicots	*Fabales*	*Fabaceae*	*Vigna*	*Vigna angularis*
NC_007957.1	Eudicots	*Vitales*	*Vitaceae*	*Vitis*	*Vitis vinifera*
NC_023259.1	Eudicots	*Geraniales*	*Vivianiaceae*	*Viviania*	*Viviania marifolia*
NC_013086.1	Lycopodiophyta	*Selaginellales*	*Selaginellaceae*	*Selaginella*	*Selaginella moellendorffii*
NC_008829.1	Moiliformopses	*Marattiales*	*Marattiaceae*	*Angiopteris*	*Angiopteris evecta*
NC_014699.1	Moiliformopses	*Equisetales*	*Equisetaceae*	*Equisetum*	*Equisetum arvense*
NC_014348.1	Moiliformopses	*Dennstaedtiales*	*Dennstaedtiaceae*	*Pteridium*	*Pteridium aquilinum*
NC_016063.1	Gymnosperms	*Pinales*	*Cephalotaxaceae*	*Cephalotaxus*	*Cephalotaxus wilsoniana*
NC_009618.1	Gymnosperms	*Cycadales*	*Cycadaceae*	*Cycas*	*Cycas taitungensis*
NC_026301.1	Gymnosperms	*Gnetales*	*Gnetaceae*	*Gnetum*	*Gnetum gnemon*
NC_024022.1	Gymnosperms	*Pinales*	*Cupressaceae*	*Juniperus*	*Juniperus monosperma*
NC_021456.1	Gymnosperms	*Pinales*	*Pinaceae*	*Picea*	*Picea abies*
NC_011153.4	Gymnosperms	*Pinales*	*Pinaceae*	*Pinus*	*Pinus contorta*
NC_023805.1	Gymnosperms	*Pinales*	*Podocarpaceae*	*Podocarpus*	*Podocarpus lambertii*
NC_016065.1	Gymnosperms	*Pinales*	*Cupressaceae*	*Taiwania*	*Taiwania cryptomerioides*

### Construction of non-redundant chloroplast reference

Using the CpGenome of *Arabidopsis thaliana* as the initial reference sequence (as a database sequence), other selected CpGenomes (as query sequences) were aligned to the database sequence by BLAST+ v2.2.25 software with default parameters. The sequences with more than 90% identity were masked from the query sequences. Then, the resulting sequences were subjected to a secondary round masking of redundant sequences, which were identified by an all-against-all BLAST+. Finally, a non-redundant chloroplast reference, as a pan-chloroplast genome (pan-CpGenome), was obtained by iterative analysis. Sequences with high similarity (> = 90%) were masked with “Ns”, and others were highly divergent sequences in the pan-CpGenome (Supplementary File F1). The visualization of the alignment of 98 CpGenomes to *Arabidopsis thaliana* CpGenome was conducted by BLAST Ring Image Generator (BRIG V0.9) [10] with default parameters.

### Universal probes designed for bamboo CpGenomes

The regions of the pan-CpGenome sequences which have not been masked to “Ns” were extended by 40 bp on both sides for the design of the probes. Each region was divided into *K*-mers of 90 bp in length and the melting temperatures of the *K*-mers were calculated [11]. A comprehensive score of uniqueness, frequency, melting temperature, and GC content was calculated for each probe by Primer3 v2.4.0 [12]. The probes with the highest comprehensiveness scores were selected in 20 bp window and slid along the target region at the fixed interval. For ensuring high coverages of the probe sequences in the target region, the target region was covered at least 2 times by these selected probes. Finally, a total of 180,519 DNA oligonucleotides were synthesized by a CustomArray B3 Synthesizer (CustomArray, Washington, DC, USA) according to the manufacturer’s instructions and dissolved in 10× TE buffer (pH = 8.0).

### Taxa sampling

All sampled species covering more than 30 genera (Supplementary Table S4) were collected in spring 2015 and 2016 under the permission of four main bamboo gardens in China: (1) Taiping base of ICBR: N:30°20′57.03″, E:118°01′30.21″, 150 M, (2) WangJianglou Park, Chengdu: N:30°37′54.85″, E:104°05′23.84″, 150 M, (3) Yunnan Pu′er Asia Bamboo and Rattan Exposition Garden: N:22°41′24.67″, E:100°56′26.51″, 1000 M, and (4) BaiMa base of Nanjing Forestry University: N:31°36′35.62″, E:119°10′34.29″, 50 M. During the sampling process, identification services of bamboo samples were provided by related taxonomists at each bamboo garden. Totally, 358 bamboo samples, mainly from young leaves, were collected. All samples were frozen in liquid nitrogen immediately and were preserved in ultra-low temperature refrigerator at − 80 °C, followed by DNA extraction.

### DNA extraction and target enrichment sequencing for bamboos

A total of 358 woody bamboo samples were sampled and sequenced in this study (Supplementary Table S4), as a practical application of target enrichment sequencing and an evaluation of the capture efficiency. Genomic DNA from each sample was extracted using the CTAB method and fragmented to a peak size of 200 bp using a Covaris E220 sonicator (Covaris, Woburn, Massachusetts, USA), followed by the end-repair, addition of base “A”, and adapter ligation. DNA fragments of the desired size (200 bp) were selected on an agarose gel and hybridized to the probes for 72 h. The probes captured DNA fragments were recycled by magnetic beads coated with streptavidin, which interacted with the biotin on the probes to wash away the uncaptured DNA fragments.

The captured DNA fragments were sequenced on the BGISEQ-500 platform at Beijing Genomics Institute, Shenzhen, China. High-quality reads ranging from 1 Gb to 9 Gb with 100 bp paired-end were acquired for each sample. For data preprocessing, we illuminated our method in a flow chart (Supplementary Figure S1). SOAPfilter (v2.2) [13] was applied to remove low-quality reads and adaptors in the following criteria (1) reads with > 10% base of N; (2) reads with > 40% of low-quality reads (value <=10); (3) reads contaminated with adaptors and produced by PCR duplication. A CpGenome of *Phyllostachys edulis* (downloaded from NCBI, accession number: HQ337796.1) was used as a reference for assembly using MITObim (V1.8) [14]. In this way, we finally recovered the complete CpGenomes of all 358 samples. Additionally, the plastid genomes were annotated in the current standard web-based program DOGMA [15] (http://dogma.ccbb.utexas.edu/,).

### Phylogenetic analysis of woody bamboos

We downloaded previously published CpGenomes of 69 bamboo species from NCBI (released May 2020) to amplify the sampling of the species tree (Supplementary Table S5). Redundancy sequences were removed, resulting in 412 non-redundant bamboo CpGenomes (Supplementary Table S6). The CDS sequences of each gene family were aligned using MAFFT (V7.017) [16] with default parameters based on the corresponding protein sequences, and then sequences were concatenated to produce 54,078 nucleotide positions. A maximum likelihood (ML) species tree was constructed with IQ-TREE (V1.6.12) [17] with parameters: -m MFP, −B 1000, −bnni, −alrt 1000.

### Sharing the bamboo CpGenome dataset

All 358 woody bamboo CpGenomes provided in Supplementary Table S4 were deposited in China National GeneBank (CNGB) (https://db.cngb.org/blast/blast/blastn/), with the database named “Chinese Bamboo Database”. The CNGB developed BLAST+ (version 2.6.0) service to allow public searches against the bamboo CpGenomes.

## Results

### Development of universal chloroplast probes for bamboos

**Fig. 1:**
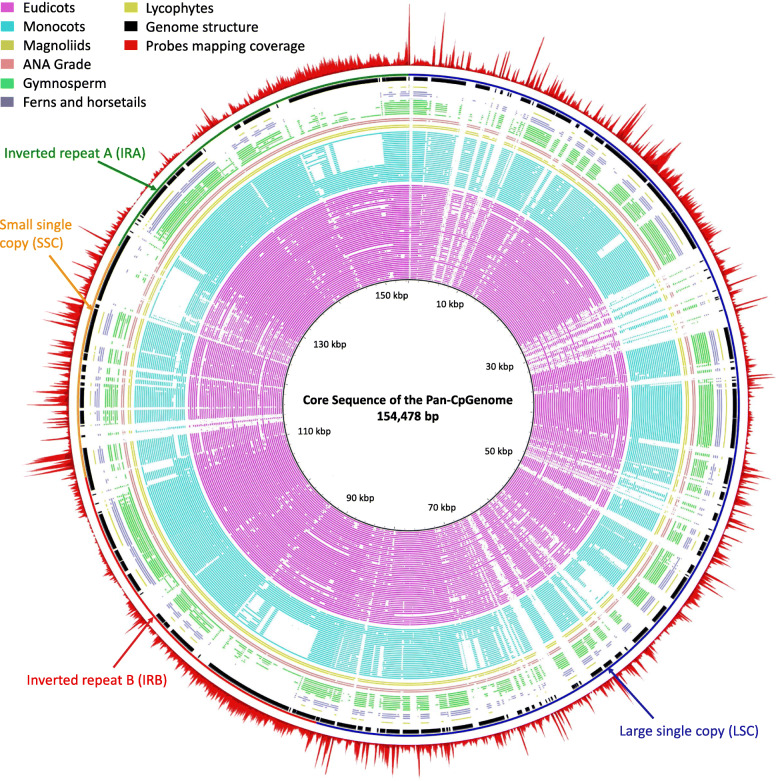
The circle of alignment and depth sketch of a core CpGenome by BRIG. The CpGenome of Arabidopsis thaliana with a length of 154,478 bp was used as the core sequence of pan-genome. Please see the details for Methods. The inner circles show the alignment of 7 clade CpGenomes to *A. thaliana* using BLAST+. The black circle indicates gene positions, and adjacent colorful circles manifest the genome structure of *A. thaliana*. Based on DOGMA, the CpGenome was divided into four sections: Inverted Repeat A (IRA), Small Single Copy (SSC), Inverted Repeat B (IRB), and Large Single Copy (LSC). The outer circle shows the depth of the probes mapping to *A. thaliana*

**Fig. 2:**
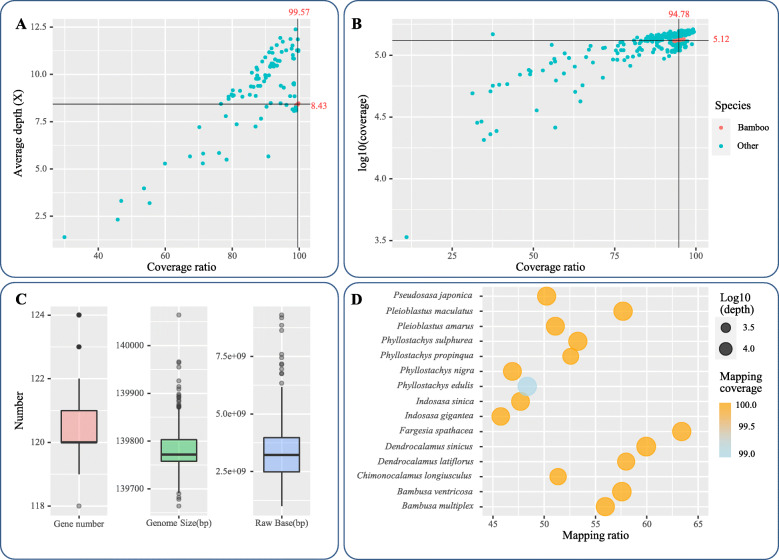
Evaluation of the pipeline performance in woody bamboos. **a** A dot plot provides the average depth (×) and coverage ratio of the 99 plant CpGenomes used to design the probes. The red and blue dots represent bamboos and other plant species, respectively. The black lines represent the average depth (×) and a coverage ratio of the bamboo species. **b** A dot plot provides log10(cover length) and the coverage ratio of the 468 plant CpGenomes used to evaluate the probes. The red and blue dots represent bamboos and other plant species, respectively. The black lines represent log10(cover length) and the coverage ratio of the bamboo species, respectively. **c** A box plot of gene number, genome size, and raw bases (bp) of the sequenced bamboos CpGenomes in this study. **d** Evaluation of mapping and coverage of the probes compared to the in-house and released bamboo CpGenomes. The mapping ratio represents the proportion of reads obtained by the probes aligned with the released bamboo CpGenomes. Mapping coverage represents the proportion of the assembled CpGenomes based on the probes aligned with the released bamboo CpGenomes

From the 3654 CpGenomes collected from NCBI, 567 high-quality CpGenomes were selected for probe development and divided into two datasets, with 99 CpGenomes for probe design and 468 CpGenomes for probe evaluation. Considering the applicability and robustness of the probes designing for bamboos, and the diversity of CpGenomes, the 99 CpGenomes were selected from different families. Details of the related methods were provided in Supplementary Figure S1. A 15 Mb pan-CpGenome was assembled based on the alignment to *Arabidopsis thaliana* (Supplementary File F1). The comparison analysis showed the CpGenomes had great variations across species (Fig. 1). Lycophytes CpGenome showed the greatest gaps in the alignment, followed by Ferns, Horsetails, and Gymnosperm. Eudicots and some of Monocots had the highest integrity of CpGenomes. Compared to Eudicots, some of Monocots, Gymnosperm, Ferns, Horsetails, and Lycophytes had large gaps at 146–150 kb, 124–129 kb, and 88–92 kb. According to the mapping depth, the depth of probe coverage at 100–110 kb, 35–42 kb, and 130–140 kb were rather lower than at other sites. For evaluating the quality of the pan-CpGenome, we calculated the coverage of the probes designed for the 99 complete CpGenomes. Alignment with the 99 reference CpGenomes showed an average coverage of 88.2% and an average base depth of 9.04×. In bamboos, the corresponding average coverage and average base depth were 99.6% and 8.43×, respectively (Fig. 2a).

A total of 180,519 (21,842,799 bp) probes, covering 92.04% of target regions, were designed and showed high consistency in their theoretical melting temperatures and GC contents (Supplementary Table S3). The probes sequences were available in Supplementary File F2. All the designed probes had excellent uniqueness, with an average 1 time while being aligned with the pan-genome. The probes were mostly distributed in the range of 70–80% melting temperatures and 30–40% GC content (Supplementary Figure S3). To assess the broad spectrum of the probes, the BLAST+ program was employed to align the probes to the 468 complete CpGenomes for evaluating the probes. The average coverage ratio in the 468 complete CpGenomes was 90.54% (Supplementary Table S8). In bamboos, the coverage ratio was all over 93.00%, with an average coverage of 94.78% (Fig. 2b and Supplementary Table S8). Moreover, some orders such as Magnoliales, Pinales, Poales also had high coverage.

### Probe-based targeted enrichment and assembly of bamboo CpGenomes

A total of 358 fresh woody bamboo samples collected from China were included (Supplementary Table S4) and used to evaluate capture efficiency. A total of 1G–9G raw reads were obtained, and low-quality reads and adaptors were filtered in data preprocessing (Fig. 2c and Supplementary Table S9). Clean and high-quality reads were used for reference-guided assemblies by MITObim and recovered nearly complete CpGenomes for the 358 bamboo species. The assembled CpGenomes ranged from 139,664 to 140,064 base pairs (bp), and the LSC regions varied from 83,496 bp to 83,845 bp in length (Supplementary Table S9). The CpGenomes were annotated with approximately 121 genes, including around 113 unique genes encoding 80 proteins, 4 ribosomal RNAs, and 29 transfer RNAs, exhibiting a higher degree of conservation.

We detected 15 overlapped bamboo CpGenomes that were present in both the in-house and published data (Fig. 2d). To assess the target enrichment, we mapped the raw reads to the corresponding CpGenome released previously and compared assembled bamboo CpGenome to corresponding released ones. The results showed more than 45.77% in average of the raw reads from in-house bamboo CpGenomes can be mapped to the corresponding published CpGenomes, and the mapping depth was higher than 1200×. Alignment with the published CpGenomes, the coverage of assembled CpGenomes was greater than 98.59% (Fig. 2d and Supplementary Table S10).

### A phylogenomic relationship based on 412 bamboo CpGenomes

**Fig. 3:**
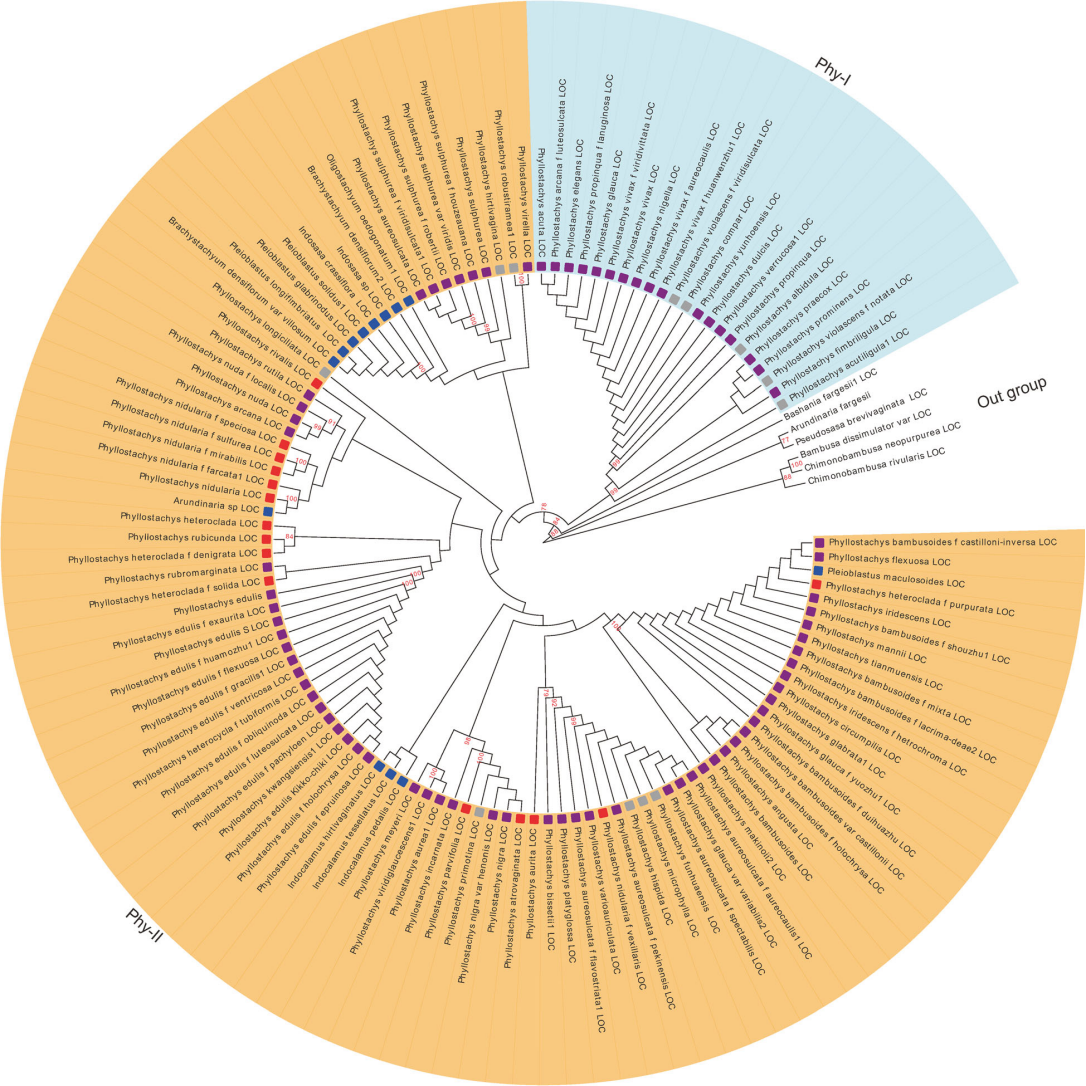
A species tree of *Phyllostachys* clade based on 76 chloroplast genes. The species tree divided into 2 parts, which labeled with different background colors. Numbers at the node indicated the bootstrap values and bootstrap values lower than 80 were concealed. The red, purple, grey, and blue blocks in the tree represented P. sect. *Heteroclada* species, P. sect *Phyllostachys* species, unlabeled and non-Phyllostachys species, respectively. Name with ‘LOC’ stands represented newly sequenced sequences in this study

For comprehensively collecting bamboo CpGenomes, 69 bamboo CpGenomes from NCBI were acquired, resulting in a total of 412 non-redundant bamboo CpGenomes after removing redundancy (Supplementary Table S6). We reconstructed a phylogenetic tree of bamboos based on the concatenated sequences of 76 protein-coding genes in the 412 bamboo CpGenomes. Phylogenetic analyses supported the relationship of (Arthrostylidiinae (Bambusinae, Olyreae)). We classified different clades in the phylogenetic tree based on previous studies [18, 19]. The pattern of (XI((VIII, IV)VI)((IX, III) (VII, V))) was provided in Arthrostylidiinae (Supplementary Figure S4). Most of the newly sequenced species distributed in Clade V, Clade VI, and Clade Paleotropical. Clade XI (*Ampelocalamus calcareus*) was the earliest diverging Arthrostylidiinae species. The *Phyllostachys* was a representative genus in bamboo, with the clade embed into Clade V, which was the sister clade of *Bashania fargesii*. There are some non-*Phyllostachys* species were found in *Phyllostachys* genus clade. The *Phyllostachys* genus clade was divided into two groups based on the phylogenetic tree. *Phyllostachys edulis*, the most planted bamboo in China, distributed in Phy-II (Fig. 3). The sequences from NCBI clustered with corresponding in-house sequences. For example, *Phyllostachys edulis* sequence from NCBI clustered with in-house sequences of *Phyllostachys edulis* f epruinosa*, Phyllostachys edulis* f exaurita*, Phyllostachys edulis* f flexuosa*,* et al.

### China Bamboo database in CNGB

The data that support the findings of this study have been deposited into CNGB Sequence Archive (CNSA) [20] of China National GeneBank DataBase (CNGBdb) [21] to facilitate the accumulation of knowledge on bamboo phylogeny. Researchers can download the raw data and assembled CpGenome sequences from CNGB through Project ID: CNP0000502 (https://db.cngb.org/search/project/CNP0000502/). Moreover, researchers can search for all assembled bamboo plastid genomes in this study through web-based BLAST+ service (https://db.cngb.org/blast/). The available plastid genome sequences of bamboos and the corresponding BLAST+ server can promote researchers to explore the complex and elusive history of bamboo evolution.

## Discussion

### CpGenome provides an essential resource for plant evolution

As an essential component of plant organelles and photosynthesis organs, chloroplasts have a simple structure, the small genome size (~ 110–165 kb) containing ~ 90–110 protein-coding genes [22] and highly conserved gene region across species, due to their non-recombinant, haploid and uniparentally [23]. The genomic characterization of various aspects of chloroplasts has led to an important role in the research of plant origin, evolution and phylogenetic analysis relationship between different plant species [24, 25]. Many studies had been reported using chloroplast genes to construct phylogenetic trees of plants. For example, Jansen et al [26] used 81 chloroplast genes to estimate relationships among the major angiosperm clades; Saarela et al [27] found weak support for *Amborella* as the basal-most angiosperm lineage using 17 plastid genes and the nuclear gene phytochrome C (*PHYC*). With the deepening of chloroplast research, more and more researchers are focusing on the complete chloroplast sequence [28–30]. Kane et al [31] suggested that the whole CpGenome could serve as an ultra-barcode for identifying plant varieties.

### Hybridization-based probes for target enrichment in large-scale CpGenome sequencing

Chloroplast DNA can be traditionally acquired by the sucrose gradient centrifugation method [6] or the high salt method [7]. Another method was to amplify the entire chloroplast DNA from the whole cellular DNA base on a long PCR technology by primers, which were designed on conserved sequences [8]. These methods were not suitable for large-scale samples due to the large amount of labor and material resources required to obtain chloroplast DNA, and the labor-intensive method used to prepare chloroplast DNA. Chloroplast reads also can be identified from WGS reads by aligning the WGS data with the reference CpGenome. It is a demanding bioinformatics technique and requires a closely related reference CpGenome. The method was not suitable for the species that are not closely related or have poor quality reference genome sequences. Moreover, to assemble only CpGenome based on this method, a great deal of useless sequencing data was thus generated, consuming much of the sequencing capacity and reducing the efficiency of parallelly chloroplast sequencing, since the chloroplast DNA sequencing data represents only a small fraction of WGS. Therefore, most existing methods for obtaining DNA and sequencing data suitable for whole CpGenomes cannot meet the needs of large-scale CpGenome sequencing, greatly limiting and hindering the in-depth research of plant genetics and evolution.

Target enrichment before sequencing is a useful method that allow for in-depth analysis of specific portions of the genome. Moreover, a group of universal probes covering whole CpGenome in a tribe species can make target enrichment strategy exert it’s advantages. Large scale CpGenomes target enrichment by universal probes can provide cost-effective, high density, and high coverage.

### Efficiency target enrichment and comparative analysis of CpGenomes for different clades

More than 3000 chloroplast genomes have been released recently [32], since the first reported sequencing of the complete CpGenome of *Nicotiana tabacum* [33]*.* We chose the 99 representative CpGenomes, including 6 bamboo CpGenomes from 3654 CpGenomes published to design probes. These vascular plants included 7 clades (Lycopodiophyta, Moiliformopses, Gymnosperms, Basal angiosperms, Monocots, Eudicot, and Magnoliidae), belonging to 57 families and 40 orders. The alignment of the CpGenomes of 7 clades to *Arabidopsis thaliana* CpGenome may show the CpGenome structure variation during evolution and indicating differences among different clades (Fig. 1). Structure variation indicated the pan-CpGenome derived from CpGenomes of distinct clades was essential for constructing greater applicability of pan-CpGenome with more divergent sequences. In 146–150 kb, 124–129 kb, and 88–92 kb, Poaceae had alignment gaps compared to the rest of Monocots, ANA grade, Magnoliids, and Eudicots. Moreover, Ferns, Horsetails, Gymnosperm, and Lycophytes indicated fragment sequences at the corresponding positions. It may suggest the corresponding CpGenome regions completed in angiosperm during evolution and uniquely lost in Poaceae after Angiosperm. However, the phenomenon should be further tested on the basis of broad-spectrum reference and amplification samplings.

In pan-CpGenome construction, unique sequences were selected, and the final pan-CpGenome size was ~ 15 Mb. A total of 180,519 probes were designed and synthesized using a new hybridization-based approach to enrich chloroplast DNA fragments. Evaluation of the quality of the probes and pan-CpGenome showed a high mapping ratio, which was stable and efficient in bamboo CpGenomes. Besides bamboos, the amplified plant CpGenomes expanded variational sequences and universality of the probes in the pan-genome construction step. Thus, the probes also had high mapping rates in some orders, such as Malvales, Rosales, Pinales and Poales, et al, and indicated the applicability of the probes in these clades. Conversely, lower mapping rates were found in Nymphaeales, Solanales, Schizaeales, Lamiales, *et al*, which may due to inadequate and poor corresponding CpGenomes materials in pan-Genome constructing. It can be solved by amplifying corresponding CpGenomes to expand divergent sequences in pan-CpGenome or decreasing parameter restriction. Comparing of the assembled CpGenome with its published counterparts demonstrated a mapping coverage of over 98%, further confirming the efficiency of the probes in enriching chloroplast DNA fragments. In general, this pipeline of pan-CpGenome construction, pan-CpGenome-based probes design, and CpGenome enrichment showed its performance in bamboo CpGenomes and recommended a strategy of large-scale CpGenomes acquiring to green plants.

### Bamboo CpGenomes could provide additional information on large-scale phylogenetic relationships

There are more than 500 bamboo species in China, which play significant roles in economy, ecology, culture, aesthetics, and technology [34, 35]. Bambusoideae is one of three subfamilies in Poaceae known as the BEP clade [36]. Bamboo remains one of the most challenging groups for plant taxonomists and field botanists [37], due to infrequent, incongruent, unpredictable flowering events, and diversity vegetative characters, which may result from frequent hybridization occurred in bamboos [37, 38]. As a useful strategy in phylogenetics and classification of species, phylogenetic analysis based on sequences has been performed in bamboos over the past decades. Extensive sampling and sequencing of the plastid genome has been a remarkable effort in genetic, phylogenetic, and classification analysis of bamboo. We have constructed a phylogenetic tree of 412 samples, covering more than 300 species, 40 genera, which is the largest sampling project of bamboo in China and provides a large-scale phylogenetic tree of bamboos. According to the phylogenetic tree, XI (*Ampelocalamus calcareus*) is the earliest diverging Arthrostylidiinae species, consistent with previous studies [18, 19, 39]. The phylogenetic tree supports (Arundinarieae (Bambuseae, Olyreae)) pattern, and the pattern is consistent with previous studies based on smaller-scale plastid sequences, suggesting a non-monophyletic lineage of woody bamboos [36, 40–42]. The results also showed the stability of the pattern, which may no change under amplified sampling. Differently, phylogenetic trees using nuclear sequences suggested the basal position of Olyreae in Bambusoideae and showed a monophyletic origin of the woody characteristic of bamboo [37, 43]. For clarifying the confliction, the analysis should focus on changes in gene duplications and genome structure caused mainly by multiple hybridizations in bamboo, by performing largely amplified sampling and genome-wide sequences. Additionally, there is a fundamental demand for bamboo life trees, especially in China, which has the world’s largest areas of bamboo plantation [34].

The *Phyllostachys* genus, with 59 species, is the most economically important among bamboos [44–46]. *Phyllostachys edulis* is the most significant *Phyllostachys* species, accounting for ∼73.8% bamboo-growing regions in China (4.43 million ha), and is the most abundant non-wood resource [34]. This study included 102 *Phyllostachys* CpGenome sequences, covering more than 90% *Phyllostachys* species, and provides an unprecedented opportunity to expand taxonomic knowledge of *Phyllostachys* genus. Traditionally, *Phyllostachys* genus can be divided into two groups, P. sect. *Phyllostachys* and P. sect. *Heteroclada*, based on morphological features such as inflorescences and rhizomes *et al.* [47, 48] But there is a controversy in this classification due to some in-between morphological features of two groups [44, 47]. Compared to the traditional taxonomy, the species tree we constructed exhibited different phylogenetic relationships in P. sect. *Phyllostachys* and P. sect. *Heteroclada*, specifically the two groups of species intermixed in the species tree. Incongruence between morphological taxonomy and the phylogenetic tree may be due to complex evolutionary processes or taxonomic treatments. Totally, 13 non-*Phyllostachys* species, such as *Indocalamus pedalis*, *Oligostachyum oedogonatum*, *Pleioblastus solidus, et al* were found in *Phyllostachys* genus Clade. They are all scattered in Phy-II. The existence of numerous non-*Phyllostachys* species may indicate non-monophyly of the *Phyllostachys* genus. It is supporting the non-monophyly thesis of *Phyllostachys* genus based on previous studies of plastid sequences [38, 49, 50] and conflicting with previous results based on non-genome wide nuclear sequences or morphological features [44, 47, 48]. The classification should be treated carefully because of the evolutionary complexity of bamboos. Moreover, The incongruence between plastid and nuclear gene phylogenies in Arundinarieae was found in the previous study [18]. Though the species tree we constructed supports more than 90% species coverage of *Phyllostachys*, the taxonomy of *Phyllostachys* clade should be further tested within the phylogenies based on genome-wide nuclear genes.

## Conclusions

A practical and large-scale approach to CpGenome acquisition will promote plant genetics and phylogenetics. We recommend a universal probe-based CpGenome enrichment pipeline, which successfully applied to bamboo CpGenomes, and 358 woody bamboo CpGenomes were acquired. Moreover, the universal probes we designed for bamboo exhibited a broad spectrum, which may also be applicable in Magnoliales, Pinales, Poales et al. We also reconstructed a phylogenetic tree of bamboos in China based on CpGenomes which supported the non-monophyly of the genus *Phyllostachys*. For promoting evolution, phylogenetic and population studies, we uploaded the sequences to CNGB to provide a BLAST+ server. For further research, we will explore many divergent hotspot regions associated with repeat sequences of LSC regions, such as tRNA clusters, which can be used as genetic markers for phylogenetic studies.

## Supplementary Information

**Additional file 1: Figure S1.** A flow chart provided for data analysis in this study.

**Additional file 2: Figure S2.** A phylogenetic tree constructed by 567 complete chloroplast genomes. These species span the phylogenetic diversity of 7 major clades including 40 order and 57 family. The details were provided in Supplementary Table S2.

**Additional file 3: Figure S3**. The density plot of melting temperatures and the barplot in GC content in probes. (A) The density of melting temperatures. (B) The barplot of GC content.

**Additional file 4: Figure S4.** An unprecedented precise phylogenetic tree of China bamboos based on 412 in-house and released bamboo chloroplast genomes. The words in red represent tree tribes in bamboos. The number at the node indicates the bootstrap value. The words in bold represent different subtribes in bamboos. The Phyllostachys Clade was underlined by light blue background.

**Additional file 5: Supplementary Table S1.** 3654 species used in probe design. **Supplementary Table S2.** The table of 568 representative species ID. **Supplementary Table S3.** Evaluation of designed probe. **Supplementary Table S4.** The bamboo species in this study. **Supplementary Table S5.** The table of bamboo plastid sequences from NCBI. **Supplementary Table S6.** The species used for species tree construction. **Supplementary Table S7.** Coverage and depth analysis of pan-CpGenome. **Supplementary Table S8.** Align the probes to 468 complete chloroplast genomes, which did not contain the sequences used to design the probes. **Supplementary Table S9.** The statistics of assembled plastid sequences after redundancy removing. **Supplementary Table S10.** Evaluation for target enrichment and assemblies.

**Additional file 6: Supplementary File F1. Pan-genome file.** The file can be opened as txt file.

**Additional file 7: Supplementary File F2. Probe sequences file.** The file can be opened as txt file.

**Additional file 8: Table X.** The genes used in species tree construction.

## Data Availability

The datasets supporting the conclusions of this article are available in the CNGB repository, https://db.cngb.org/search/project/CNP0000502/.

## Abbreviations

CNGB: China National GeneBank
CpGenome: Chloroplast genome
IR: Inverted repeat
IRA: Inverted Repeat A
IRB: Inverted Repeat B
LSC: Large single-copy region
NGS: Next-generation sequencing
pan-CpGenome: Pan-chloroplast genome
PHYC: Phytochrome C
SSC: Single-copy region
WGS: Whole genome sequencing
