# Supplementary figures and images for: Targeted enrichment of novel chloroplast-based probes reveals a large-scale phylogeny of 412 bamboos
Source: BMC Plant Biol. 2021 Feb 5;21:76. doi: 10.1186/s12870-020-02779-5 (PMC7863319; doi:10.1186/s12870-020-02779-5)

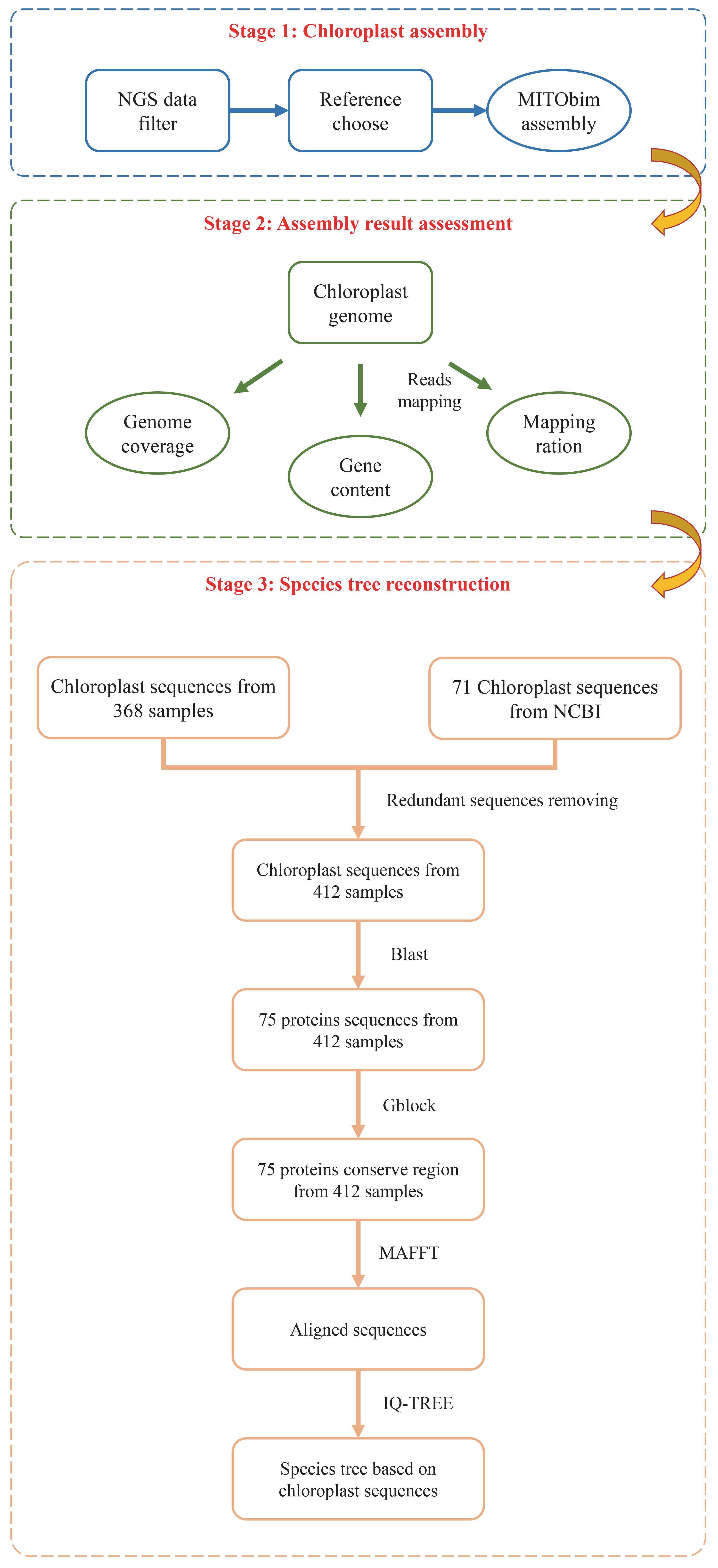

Supplement: Supplementary file 1 — Additional file 1: Figure S1. A flow chart provided for data analysis in this study. [file 12870_2020_2779_MOESM1_ESM.tiff]

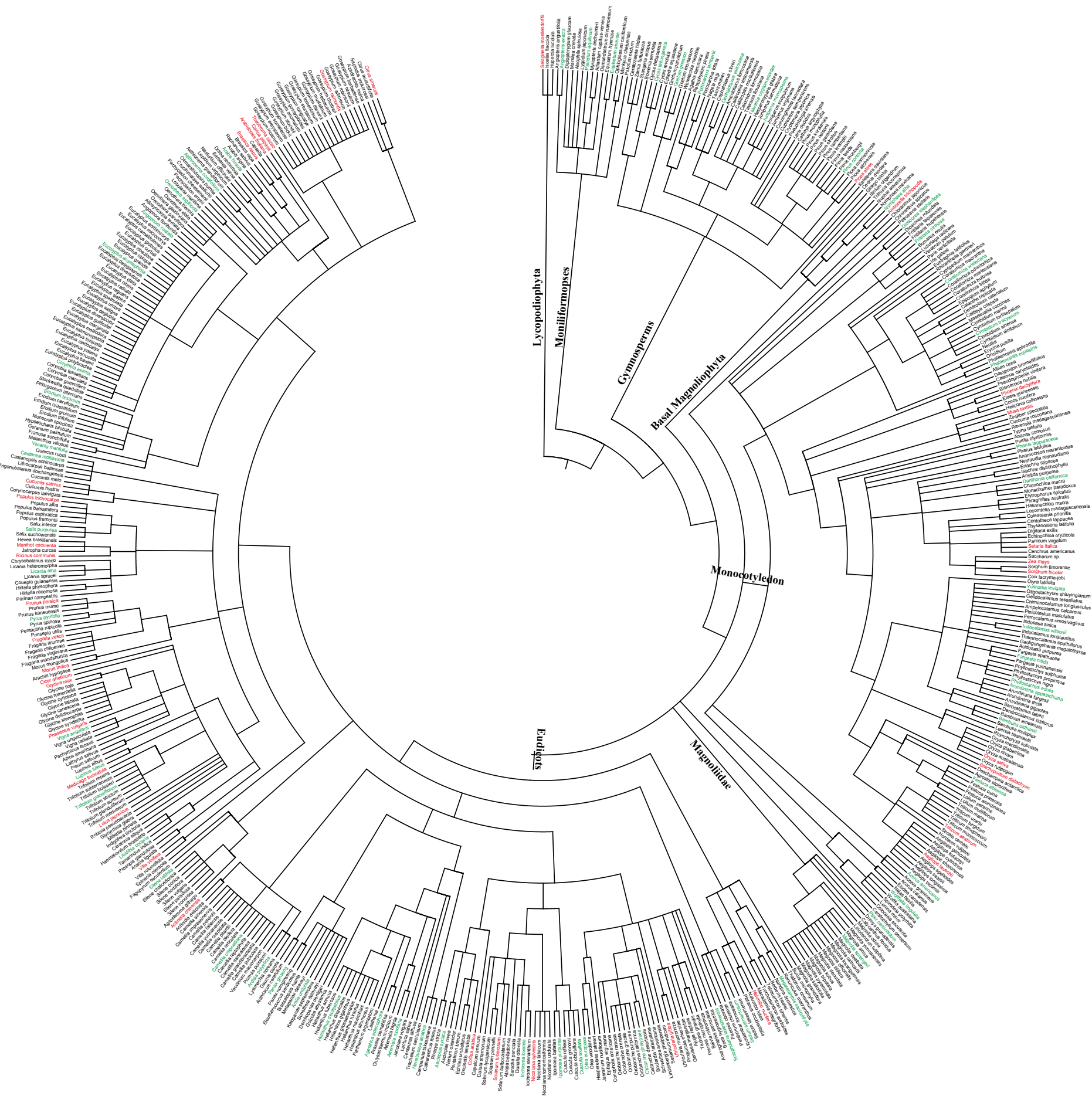

Supplement: Supplementary file 2 — Additional file 2: Figure S2. A phylogenetic tree constructed by 567 complete chloroplast genomes. These species span the phylogenetic diversity of 7 major clades including 40 order and 57 family. The details were provided in Supplementary Table S2. [file 12870_2020_2779_MOESM2_ESM.pdf]

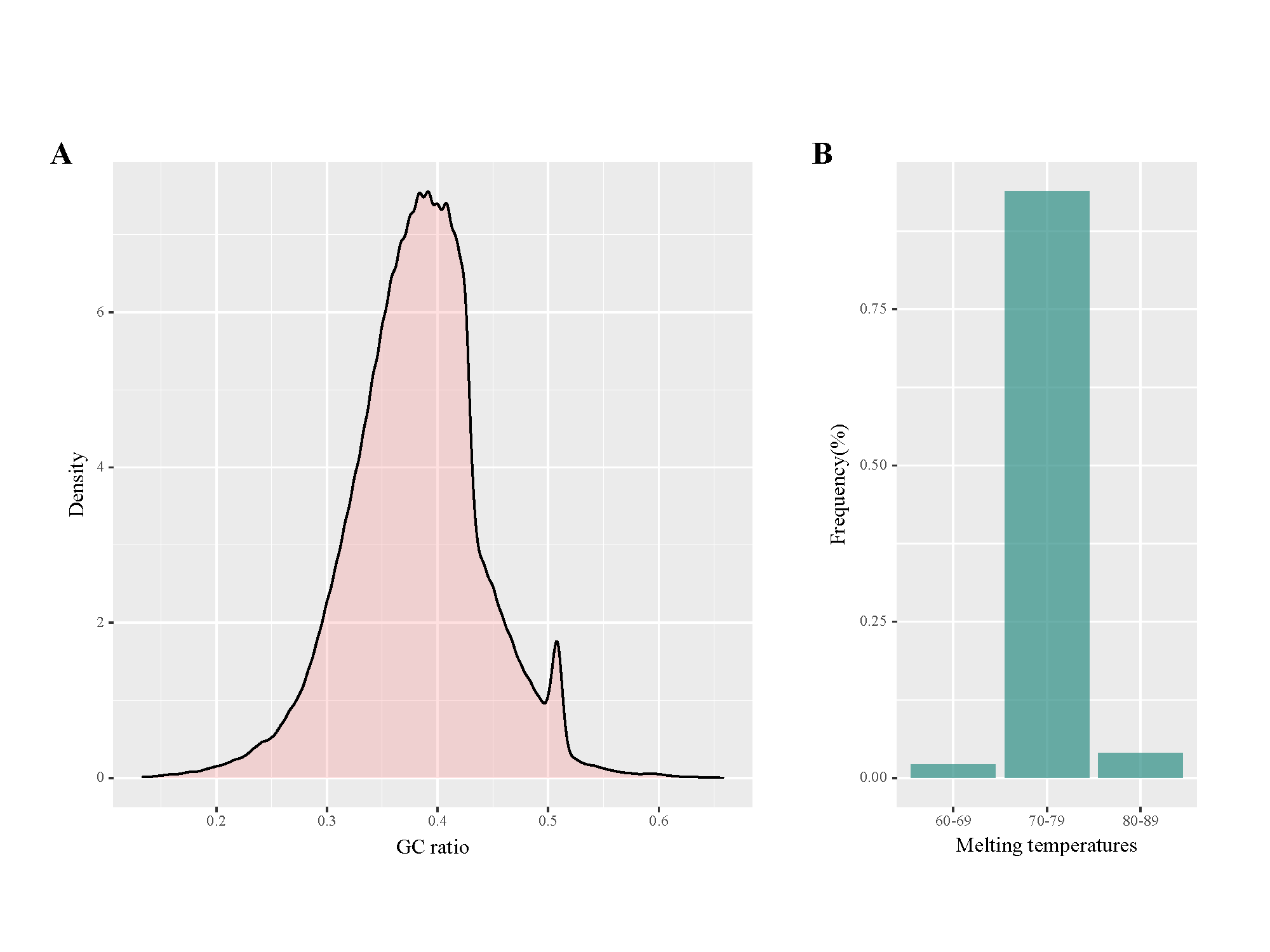

Supplement: Supplementary file 3 — Additional file 3: Figure S3. The density plot of melting temperatures and the barplot in GC content in probes. (A) The density of melting temperatures. (B) The barplot of GC content. [file 12870_2020_2779_MOESM3_ESM.tiff]

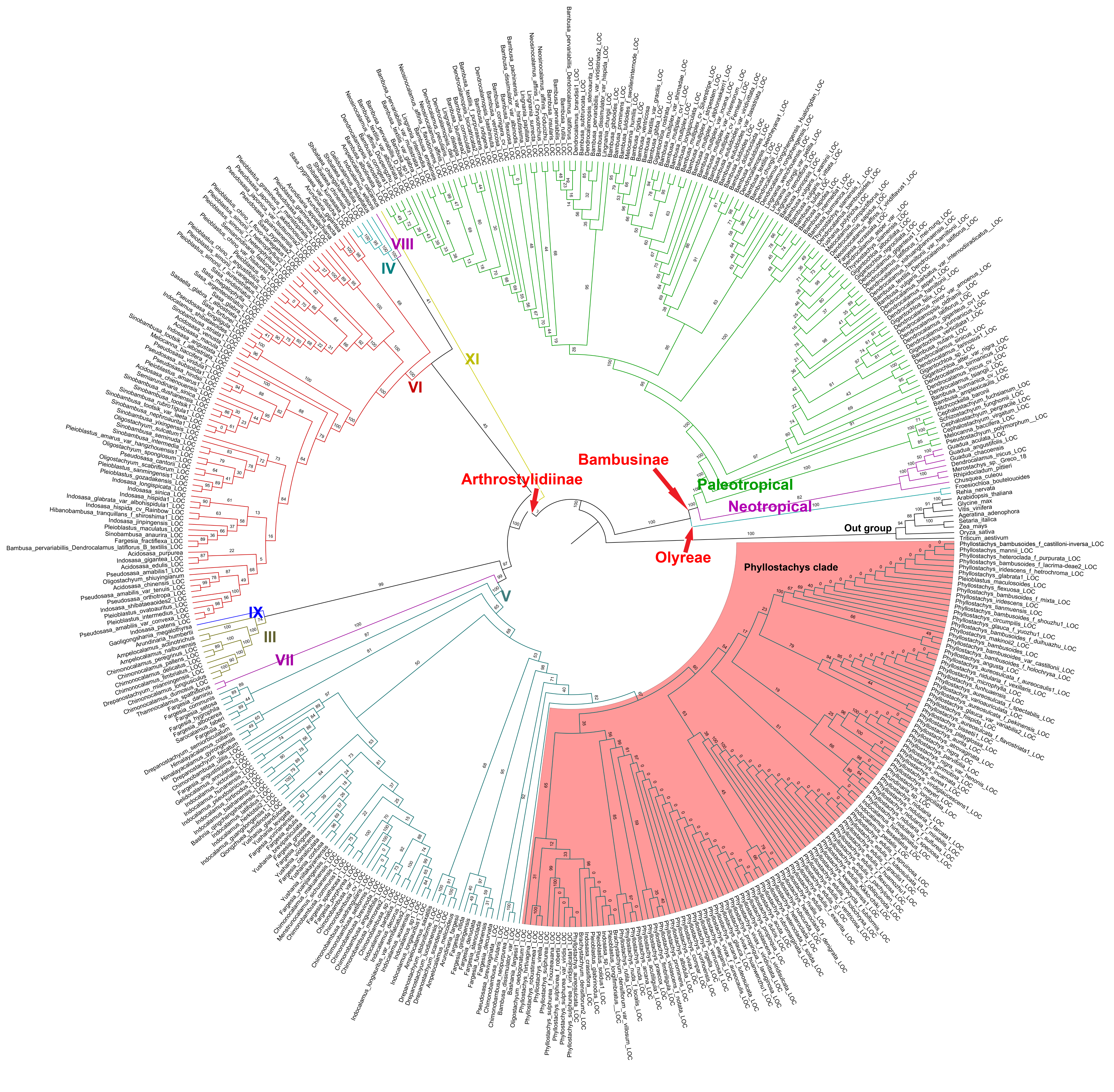

Supplement: Supplementary file 4 — Additional file 4: Figure S4. An unprecedented precise phylogenetic tree of China bamboos based on 412 in-house and released bamboo chloroplast genomes. The words in red represent tree tribes in bamboos. The number at the node indicates the bootstrap value. The words in bold represent different subtribes in bamboos. The Phyllostachys Clade was underlined by light blue background. [file 12870_2020_2779_MOESM4_ESM.tiff]
